# Acknowledgement to Reviewers of *Viruses* in 2019

**DOI:** 10.3390/v12010119

**Published:** 2020-01-17

**Authors:** Viruses Editorial Office

**Affiliations:** MDPI, St. Alban-Anlage 66, 4052 Basel, Switzerland

The editorial team greatly appreciates the reviewers who have dedicated their considerable time and expertise to the journal’s rigorous editorial process over the past 12 months, regardless of whether the papers are finally published or not. In 2019, a total of 1164 papers were published in the journal, with a median time to first decision of 18 days and a median time from submission to publication of 37 days. The editors would like to express their sincere gratitude to the following reviewers for their generous contribution in 2019:

A. Sally DavisKatja C. WolthersAarthi NarayananKato TatsuyaAartjan Te VelthuisKatri JalavaAbdul A. WaheedKatsumi MizutaAbelardo Silva-JúniorKatsuro HagiwaraAbhay PS RathoreKaty GaythorpeAbhijeet A. BakreKay FaabergAbigail B. DiackKazuhiro IshibashiAbimbola KolawoleKazuo NishigakiAdam GeballeKei SatoAdam GrundhoffKeiichiro MatsukuraAdam HumeKeith ChappellAdam LauringKelli L. BarrAdam R. HerspergerKelly LeeAdam TaylorKen KomatsuAdelaide MilaniKen LemonAdrian Alejandro ValliKen LiuAdrian J GibbsKen RosenthalAdriana DelfraroKendra AlfsonAhmed Abd El WahedKenji S. NakaharaAhmed ArslanKenneth A. StaplefordAhmed HadididKenneth LundstromAhmed HusseinKenta SHIMIZUAjeet KaushikKen-Taro SekineAke LundkvistKentaro TohmaAkihiko MaedaKerry MauckAkiko KashiwagiKeun-Seok SeoAkira NakanishiKevin CoombsAkira OnoKevin D. PavelkoAlain DublanchetKevin FonsecaAlan BarrettKevin LamkiewiczAlan FauconnierKevin SokoloskiAlan G. GoodmanKim BlasdellAlan GoodmanKim GreenAlan ReinKim J. HasenkrugAlana ThackrayKinjiro MorimotoAlban RametteKirsten WhiteAlbert RizvanovKirsty ShortAlberta AzziKjell SergeantAlberto A. AmarillaKlaus HedmanAlberto BosqueKlaus StrebelAlbieKoen K.A. Van RompayAlec GerryKoenraadAlec J. HirschKoh FujinagaAlejandro Marin-LopezKonstantin AlekseevAlejandro VallejoKosuke MurakamiAlessio LorussoKouacou KonanAlex ComptonKow-Tong ChenAlex GreenwoodKrishanu RayAlex M MurphyKristen BernardAlexander GultyaevKristin M WhitworthAlexander KhromykhKristine WylieAlexander L. GreningerKristine YoderAlexander LukashevKrzysztof TrederAlexander M ShestopalovKuan Rong ChanAlexander MalogolovkinKui YangAlexander N. FreibergKumiko YoshimatsuAlexander PasternakKurt E. WilliamsonAlexander V IvanovKurt GustinAlexander ZakhartchoukKurtis J. C. PeiAlexandra SchuetzKwang-Zin LeeAlexandre LeitãoKwinten SliepenAlexandre MachadoKylene Kehn-HallAlexei KorennykhKyung H ChoiAlexis M. KalergisKyung-Soo InnAli AbdallahLalita PriyamvadaAlí AlejoLara HerreroAli ZaidLara TavoschiAlice FusaroLarisa PoluektovaAlicia McLuckieLars FieselerAlicja WegrzynLaura BenitezAllan J. ZajacLaura BergnerAllan Randrup ThomsenLaura BrutscherAllen GrollaLaura GoodmanAllison AbendrothLauren McDanielAllison Ballandras-ColasLaurence JossetAlyson KelvinLaurent BigarréAmanda E. BayleyLaurent DebarbieuxAmelia K. PintoLaurent PerezAmesh AdaljaLee CohnstaedtAmilton De MelloLeon GrayferAmin S. AsforLeonardo SustaAmir SteinmanLeonid KulakovAmirbabak Sioofy-KhojineLes DomierAmy B. RosenfeldLeslie ChandrakanthaAmy HudsonLester J. PérezAmy MacNeillLester M. ShulmanAmy SchuhLeticia BotellaAna DomenechLeticia RuizAna MorenoLeyi WangAna ReisLia Van Der HoekAna Rita CostaLíbia Zé-ZéAna Rita Gonçalves CabecinhasLidia Lasecka-DykesAna Silvia Gonzalez-ReicheLigan WuAna-Belen BlazquezLiliana M. FinkielszteinAnan JongkaewwattanaLimin ZhengAnastasia N. VlasovaLinda BrunotteAnbu Kumar KaruppannanLinda KingAnders NilssonLinda SaifAndor DoszpolyLindsay BlackAndre BoonstraLindsey SoldenAndré Luis Costa-Da-SilvaLindsey Van HauteAndrea CivraLing WangAndrea GamarnikLinzhu RenAndrea KroekerLisa G. NevenAndrea ZbindenLisa GralinskiAndreas GahlmannLisa LindesmithAndreas KruegerLisa NgAndres MeritsLisa Zeigler AllenAndreu Comas-GarciaLise Kirstine KvisgaardAndrew AllisonLiubov I. KozlovskayaAndrew BroadbentLivia DonaireAndrew D. MillardLivia StavoloneAndrew DalbyLogan BanadygaAndrew HendersonLorant LakatosAndrew J. HendersonLorenzo FraileAndrew LangLori R. HoltzAndrew MillardLouis Du PlessisAndrew S. BowmanLuca VaraniAndrew ShawLucía FernándezAndrew Van Den HurkLucile EspertAndrey LetarovLuděk EyerAndrzej J. GörskiLuis Alberto Gaitán-CepedaAnette Gleitze BøtnerLuis G. Gimenez-LirolaAngela CrawleyLuis M. AgostoAngela L. RasmussenLuis M. Hernandez-TrianaAngela M. Bosco-LauthLuis Martinez-GilAngela PearsonLuis Menéndez-AriasAngus WilsonLuis Perez Garcia-EstañAnice LowenLuis SarmientoAnita McElroyLukasz DziewitAnja KiparLukasz KurykAnjali JoshiLukasz RabalskiAnke C. StöhrLuwanika MleraAnna CliffeLynn EnquistAnna Gora-SochackaLynn ThomasonAnna HeitmannM. B. GottikhAnna HonkoMaarten DanialAnna Kajaste-RudnitskiMaarten Van De KlundertAnna KoromyslovaMacDiarmid RobinAnna LacastaMagdalena DunowskaAnna OrłowskaMagdalena Materniak-KornasAnna Rosa GarbugliaMagdalena PlotkaAnna SalvettiMagnus EvanderAnna Teresa PalamaraMagnus LundgrenAnna ToffanMaha ElbadryAnnamaria PratelliMahmoud NaguibAnne Balkema-BuschmannMahtab PeyambariAnne PiantadosiMaia MerabishviliAnne SimonMairene CotoAnne VanetMaja KrižnikAnnelie PlentzMaja PotokarAnnemarieke SpitzenMakoto NagaiAnnie BezierMakoto TakedaAnnie Page-KarjianMalaya Kumar SahooAnnuziata GiuseppeMalgorzata ŁobockaAnthony BoscoMałgorzata ŁobockaAnthony ChamingsMamta Chawla-SarkarAnthony FehrManabu IgarashiAnthony GriffithsManfred HeinleinAnthony MarriottManjunatha BelaganahalliAnthony SadlerManon Ragonnet-CroninAnthony SchmittManuel Martínez-GarcíaAntoine TouzéMara CironeAntoinette Van Der KuylMarc FuchsAntonella Di SottoMarc GirardAntonet M. SvircevMarc SitbonAntonet SvircevMarcela FerrésAntonieta Guerrero-PlataMarcelo SoaresAntonina DoleiMarciano PaesAntonino Di CaroMarcin LosAntonio AlcamiMarcin W. BańburaAntonio CarapelliMarco BinderAntonio PirallaMarco GoeijenbierAntonio Q. TonioloMarco IannettaAntonio Rivero-JuárezMarco Maria D'AndreaAntonito PanganibanMarcos De la PenaAntonius (Tom) OomensMargaret HOSIEAntti VaheriMargaret J. HosieApostolos BeloukasMargaret LittlejohnApril D. DavisMargaret MacDonaldApurba Krishna BhattacharjeeMargo BrintonAre BerentsenMargus VarjakAriane ToussaintMaria CapobianchiArianna CalistriMaria Cristina ThallerArianna LoregianMaria Del Mar Ortega-villaizan RomoArinjay BanerjeeMaría Eugenia DieterleArmando Arias EstebanMaría Eugenia GonzálezArnaud. BlouinMaria ForlenzaArnolfo PetruzzielloMaría ForzánArtemis RumbouMaria Joao AmorimArunava RoyMaria João CatalãoArvind VarsaniMaria K DahleAshley C. BanyardMaria KalamvokiAsit PattnaikMaria Luisa MarenzoniAssia AngelovaMaria MontoyaAssoc. Prof. Bonto FaburayMaria PajunenAsuka NanboMaria Pilar CortésAtsushi YamanakaMaria SalvatoAurelie RakotondrafaraMaria T SanchezAusra DomanskaMaria TempestaAyala RaoMaría Teresa Pérez-GraciaAyalew MergiaMaria Teresa SciortinoAyato TakadaMarian J. KillipB.A. ChinMariangela GarofaloBailey L. ArrudaMariano Sanchez-LockhartBalaji ManicassamyMaría-Teresa Pérez-GraciaBalázs HarrachMarie-Christine MaurelBapi PaharMariet C. W. FeltkampBarbara BażanówMarieta BraksBarbara BiereMariette DucatezBarbara Di MartinoMario A. PagnottaBarbara KeeMario Andres PizzornoBarbara SchmidtMario EstableBarbie Ganser-PornillosMarion EnglandBarry AtkinsonMariyana GozmanovaBarry RockxMarjorie Robert-GuroffBart HaagmansMark BrockmanBasavaprabhu PatilMark MarshBasil ArifMark ParcellsBeate M. KümmererMark StengleinBeate PiniorMark WestmanBeatriz MartinezMarkus HoffmannBeatriz NavarroMarlene DreuxBen HauseMarshall E. BloomBenedikt B. KauferMart KrupovicBenedikt KauferMarta CanutiBenjamin DewalsMarta Maria GagliaBenjamin LampMartin A. ErlandsonBenjamin R. EvansMartin BisaillonBenoit VisseauxMartin DorfBergmann RibeiroMartin DruckerBerit CarowMartin LoessnerBernadette Van Den HoogenMartin LudlowBernard MossMartin SappBernardo MainouMartin SchauflingerBernardo RodamilansMartin SchwemmleBert SchepensMartin SpiegelBhudipa ChoudhuryMartina SalákováBill WangMartine Biard-PiechaczykBill WintermantelMary HummelBin GongMarzano Shin-YiBingqian QuMarzena PazgierBinod KumarMasaharuBirgit ScharfMasahiro NiikuraBjörn F KoelMasahiro YamashitaBjorn KrenzMasaki ImaiBjörn KrenzMasamichi NishiguchiBob BlasdelMasanori KameokaBoon-Seng WongMasaru EnomotoBoris KlempaMasatoshi OkamatsuBoris M. HogemaMasfique MehediBoris PeterlinMassimo GiangasperoBożena Nejman-FaleńczykMassimo PizzatoBrad GilbertsonMassimo TommasinoBradley I. HillmanMassimo TurinaBreck A. DuerkopMathias FaureBret ElderdMathias SchemmererBrett L. HurstMatthew CollinsBrian GowenMatthew EwansBrian MurphyMatthew ParsonsBrian T. FoleyMatthew ReevesBrian WigdahlMatthew TaylorBrian WillettMatthew W. TurnbullBritta MöhlMatthias G. FischerBrittany MosherMatthias SchweizerBrunetti CRMatti JalasvuoriBruno BeaumelleMaureen LongBruno BlondelMauricio Comas-GarciaBryan C NikolaiMauricio CoppoBryce ChackerianMauricio MateuBumsuk HahmMaxine L. LinialByron CaugheyMaxine LinialC. Thomas BockMd NasimuzzamanC.A. (Christine) JansenMeaghan HancockCaijun SunMeehyein KimCameron WebbMegan BaldridgeCara PagerMelanie BrinkmannCarine Van LintMelinda BrindleyCarla MavianMelissa JonesCarla SalehMelissa MaginnisCarlos Maluquer De MotesMichael A. JoyceCarmela Garcia-DovalMichael FreseCarmen MirabelliMichael GrantCarol D. WeissMichael HuberCarolina AriasMichael JukesCarolina B. LópezMichael LagunoffCarolina ScagnolariMichael LoCaroline AlfieriMichael M. ThomsonCaroline DemeretMichael MillerCaroline LerouxMichael NevelsCarrie BattenMichael NiepmannCath ReesMichael PearsonCatherine EichwaldMichael RaheCécile BreytonMichael SchreiberCécile Lagaudrière-GesbertMichael Thomas WolfingerCésar López-CamachoMichael VeitChankyu ParkMichaela RumlováChao-Nan LinMicheal DiamondCharles GroseMichel PépinCharles H. CalisherMichel PeterschmittCharles NfonMichel RavelonandroChen LiangMichela ChiumentiCheng HuangMichele CameroCheng-Wen LinMichèle OttmannCheryl DvorakMichele TonelliChetan MeshramMichelle ArnoldChi Man TsangMichelle L. FlennikenChien-Chang LiaoMichelle TateChienjin HuangMiguel A. ArandaChih-Yen KingMiguel A. Martín-AcebesChin-Tien WangMiguel A. MartínezChithra SreenivasanMiguel Ángel Jiménez-ClaveroChiu-Ming WenMiguel E. Quiñones-MateuChloé JournoMiguel López-FerberChonsaeng KimMihnea BostinaChris LauberMikael SkurnikChris OuraMike Dyall-SmithChris RobinsonMike FlintChris UptonMike SkinnerChristian HammannMikyeong KimChristian JassoyMin WangChristian MilaniMi-Na KweonChristiane RiedelMing Kun HsiehChristie MayoMing-Shiuh LeeChristina OrrúMingxi FangChristina VarveriMingyuan HanChristine BurkardMingzhou ChenChristine CarringtonMin-Suk SongChristine GoffinetMiranda De GraafChristine JonassenMireille AnsaldiChristine PourcelMirella SalvatoreChristine SzymanskiMiriam ZagoChristoph GeislerMirko CorteseChristophe CombetMiroslav GlasaChristopher B. BuckMirosław Paweł PolakChristopher C. BroderMizuho NitaChristopher Fernandez PradaModesto Redrejo RodriguezChristopher J.R. IllingworthMohamed Abdel-HakeemChristopher L. NethertonMohammad A AlkhamisChristopher StobartMohan AmarasiriChristpher B. WhitehurstMoises Leon-JuarezChrysostomos I. DovasMolly GallagherChuan (River) XiaoMonika RadlinskaChun Shen LimMonika RinderChunfeng LiMorgan HerodChung-Chi HuMoshe Bar-JosephChunling WangMotiur RahmanCinta Prieto SuárezMotoshi Tajima Claire CrossanMudit TyagiClaire Le MarrecMuhammad MunirClara Torres BarceloMukesh KumarClaude KrummenacherMutien-Marie GariglianyClaudia R. MolinsMya Myat Ngwe TunClaudia AlteriMylene OgliastroClaudia FortunaMylène OgliastroClaudia HagedornMyraClaudia R. MolinsMyriam ErmonvalClaudia TrombettaNadine KrügerClayton CareyNagayasu EgawaClement MesedaNam-Hyuk ChoClinton JonesNancy BeerensColin ButtimerNancy C. HortonColin McInnesNaoki InoueColin ParrishNaoki TakizawaColleen JonssonNaomi ForresterConcetta CastillettiNaoyoshi MaedaCoretta Van Leer-ButerNaphak ModhiranCorie GrayNatalia CheshenkoCorinna PietschNatasha N GaudreaultCorinne Ida LasmézasNathan AhlgrenCorrales-Aguilar EugeniaNathan Burkett-CadenaCorrea-Fiz FlorenciaNathan ShererCosima ZimmermannNathaniel LandauCraig McCormickNavanietha Krishnaraj RathinamCraig MeyersNeal A. DeLucaCristian SmerdouNeda BarjestehCristiano SalataNeil MabbottCristina Ferrer-OrtaNelson Rodrigo Da Silva MartinsCurtis BrandtNereida Jiménez De OyaCynthia A. Pise-MasisonNi HongCynthia J. Koziol-WhiteNicholas A. WallaceCyprian C. RossettoNicholas BairdCyrille MathieuNicholas EyreDainius ZieniusNicholas JohnsonDaisuke HayasakaNicholas KomarDaisy W. LeungNicholas R. GuydoshDalan BaileyNicholas TaylorDamien VitourNick De ReggeDan WangNicola DecaroDana VanlandinghamNicola F. FletcherDaniel BonthiusNicola StonehouseDaniel Bourquain Nicolas Albert GilletDaniel C. NelsonNicolas BerthoDaniel CastilloNicolas BoisgeraultDaniel DepledgeNicolas LévêqueDaniel Enosi TuipulotuNicole FischerDaniel G. MeadNicole TischlerDaniel L. RowleyNiels A W LemmermannDaniel MarcNigel GrimsleyDaniel PerezNiki VassilakiDaniel RawleNikolai ProkhorovDaniel S. ChertowNikolaos GatselisDaniel TodtNina LukhovitskayaDaniela RibeiroNingguo FengDaniele FocosiNingshao XiaDanielle AndersonNisha DuggalDanielle BlondelNobuhiro SuzukiDanish MalikNoël TordoDanny BarashNolwenn JouvenetDaral J. JackwoodNora M. ChapmanDarko VončinaNoriko OgasawaraDarryl FalzaranoNorio YamamotoDavid J. SimpsonNoriyo NagataDavid A. BenfieldNuredin HabiliDavid A. DavisOk Sarah ShinDavid A. LeibOleksii SkorokhodDavid A. SchwartzOlga BelykhDavid AllenOlga KalininaDavid DuniganOlga MorozovaDavid LesbarresOlga V. MasalovaDavid MarchantOlivier LemaireDavid OrnellesOlivier ReynardDavid PasdeloupOlivier SchildgenDavid PeabodyOlympia E. AnastasiouDavid PintelOscar Roberto BurroneDavid R. HarperOsvalda De GiglioDavid ReyOurania M. AndrisaniDavid UlaetoOzgur BatumanDavid VerhoevenPablo GastaminzaDawn WeirPablo Guardado-CalvoDean MyersPanayampalli Subbian SatheshkumarDebbie McKenziePanayiota KotsakioziDeborah S. FinlaisonPaola De BenedictisDeepak ShuklaPaola VazDelia GolettiPaolo GabrieliDenys A. KhaperskyyPaolo MargariaDerek R. SteinPascale Krejbich-TrototDerek TrobaughPasquale FerranteDetlev KrugerPatricia A NuttallDetlev KrügerPatricia Agudelo-RomeroDeyin GuoPatricia DayDhananjai RaoPatricia PesaventoDiana J. M. Van Den WollenbergPatricio I. MenesesDiane AddiePatrick ForterreDiane GriffinPatrick HearingDianjun CaoPatrick McClureDiego R. HijanoPaul Andrew RowleyDimitrios ParaskevisPaul BollykyDirk JanssenPaul D. FriesenDirk JochmansPaul HickDomenico TortorellaPaul HymanDominique GarcinPaul J. Wichgers SchreurDominique Julien BurriPaul KinchingtonDominique RoussetPaul KrogstadDonald J. AlcendorPaul LambertDonald SmithPaul MossDonata HoffmannPaul RiskaDonghoon ChungPaul ShirkDong-Jun AnPaulina Niedźwiedzka-RystwejDonna NeumannPaulina Rajko-NenowDorothee BienzlePaweł P. LiberskiDouglas E. BrackneyPedro José Castro EstevesDouglas GladuePei-Wen HsiehDouwe van SinderenPeng ZhanDr. Reeta ManiPeng ZhouDrew KernPengxiang ChangDuane J GublerPengxing CaoDuane P. GrandgenettPenmetcha KumarDylan M. JohnsonPenny ClarkeE.C.M. Van GorpPenny RuddEberhard HildtPer KrygerEda AltanPeter A WhiteEdana CassolPeter G. SpeckEdmond M LinossiPeter GrovesEdson DelatorrePéter GyulaEduardo R. BejaranoPeter K. VogtEdward C. HolmesPeter NagyEdward DuboviPeter PaleseEdward EmmottPeter PelkaEdward StephensPeter TattersallEfrem LimPetr ChlandaEiji MoritaPetr KomínekEike SteinmannPetr O. IlyinskiiEkaterina StepanovaPetri SusiElena CriscuoloPhilip StevensonElena FrolovaPhilip TedburyElena PaskalevaPhilippa HawesElena ZuriagaPhilippe ColsonElina LaantoPhilippe GallayElina Lastro NiñoPhilippe GeorgelElisabetta SuffrediniPhilippe Le MercierElizabeth SpargerPia M MartensenElizabeth WhitePiergiuseppe De BerardinisElke MühlbergerPierre H. H. SchneebergerEllen ArielPierre RoquesEllen CollissonPiet Van RijnEllen KrautkrämerPilar Domingo-CalapElliot J. LefkowitzPilar García SuárezElodie Monchatre-LeroyPiotr GolecEl-Sayed Abdel-WhabPolly RoyEl-Sayed M AbdelwhabPo-Yuan KeEman AnisPranav DanthiEmanuela NorisPrasad ParadkarEmanuele MontomoliPrasun DattaEmi BarkerPrimitivo CaballeroEmilia HadziyannisPriscilla F. GerberEmiliano BiasiniPriya LuthraEmilio D'UgoPriya ShahEmma B. HodcroftPuck Van KasterenEncarnación Pérez ArtésQiang DingEnric MateuQigai HeEnrico BrugneraQingsheng LiEnrico IaccinoQinxue HuEnrique MorionesQiong ZhangEnzo TramontanoQiuwei PanEoin RyanQiya ZhangEran BacharachQiyi TangEric BarteeQiyun ZhuEric DelwartQuentin ChesnaisEric FreedRachael TarlintonEric GowansRachel PalinskiEric JanRafael DelgadoEric JohannsenRafael Kroon CamposEric MosselRafael-Jacinto VillanuevaEric WeaverRaffael NachbagauerErich R. MackowRaffaella Maria BalestriniErik De ClercqRajagopalbabu SrinivasanErik KarlssonRalph FeuerErin GoodrichRalph TrippErin M. McDonaldRandal GreggErkki TruveRandal N. JohnstonErna Geessien KroonRandal VossErwin DuizerRandall CohrsEsmaeil AmiriRandy AlbrechtEsperanza Gomez-LuciaRaphael AdegbolaEsteban A. Hernandez-VargasRaphaël GaudinEsteban DomingoRaquel HernandezEsther BullittRaquel Muñoz-MorenoEugene RyabovRasa Petraityte-BurneikieneEugenia NegredoRashid ManzoorEugenio RastelliRavi KulkarniEung-Soo HwangRebecca BrocatoEva BengtenRebecca ChristoffersonEva Böttcher-FriebertshäuserRebecca DuBoisEva HerkerRebecca LynchEva SpadaRebecca SchmidtEva VarallyayRebecca SkalskyEvelien AdriaenssensRebekah C. KadingEvžen BouřaReed F. JohnsonEwa Jończyk-MatysiakReed B WicknerEwa PlantReed F. JohnsonEwelina KrolRegina Hofmann-LehmannEyal KlementReinhard ErtlFabienne ArcherRemi N. CharrelFabio PasinRenaud MahieuxFabrizio CilloRenée Marije Van Der SluisFaizal CareemRenfeng LiFasseli CoulibalyReza KhayatFátyol KárolyRicardo ParreiraFayna Diaz-San SegundoRicardo SantosFederica Di ProfioRich BennettFederica MonacoRichard BowenFelix BroeckerRichard CalendarFelix StahlRichard G HegeleFeng ChenRichard HeinemanFeng GaoRichard KatzFeng LiRichard KormelinkFeng QuRichard KuhnFengmin LuRichard P NovickFergalRichard PlemperFernando EscriuRichard StantonFernando FerreiraRichard SuttonFernando RodriguezRichard WangFernando SpilkiRichard YanagiharaFernando Torres-PerezRik De SwartFernando Torres-PérezRimantas DaugelaviciusFilomena FonsecaRob A. GrutersFinn Skou PedersenRob HoebenFlorence AbravanelRob J. CenterFlorence BuseyneRobert A. SmithFlorencia MeyerRobert DaveyFlorian KrammerRobert HarrisonFlorian MaumusRobert HarrodFlorine E.M. ScholteRobert HertelFok Moon LumRobert J. GeraghtyFrancesca CaccuriRobert OrchardFrancesca ColavitaRobert PaxtonFrancesco BroccoloRobert PosseeFrancesco Di SerioRobert SilvermanFrancesco FelizianiRobert V. StahelinFrancini KlaumannRobert WagnerFrancisco LlorenteRobert WelliverFrancisco MalagonRobert Y.-L. WangFrancisco Murilo ZerbiniRoberta CastriconiFrancisco Rodriguez-ValeraRod RussellFranco MutinelliRoderick SlavcevFrancois HelleRodney BristerFrançois-Loïc CossetRodolfo De La Torre AlmarázFrank JenkinsRodolphe HamelFrank OechslinRoger A. MooreFrank P. ShewmakerRohit JangraFrank StubenrauchRokshana ParvinFred L. HomaRoland ZellFrédéric AuvrayRolandas MeškysFrederic LE GALRollie J. ClemFrederick J. FullerRomain VolmerFrederick KibengeRomina SalpiniFrederik SchulzRonald DijkmanFriedemann WeberRonan Le GofficFuh-Jyh JanRong HaiFun-In WangRong ZhangGábor KemenesiRosa MurilloGabriel GonzalezRoss T. BarnardGabriel HamerRoy A. HallGabriel OzorowskiRüdiger HorstkorteGabriela Pastuch-GawolekRushika PereraGabriele A. LandoltRuslan KalendarGaël BelliotRussell J. DiefenbachGaël ThébaudRyan TroyerGareth BradyRyuichi WadaGareth TrublSabine GilchGary MartySabra KleinGary R. WhittakerSabrina BertinGary TrublSabrina SchreinerGeeta UpadhyaySabrina WeissGene S. TanSaguna VermaGeoger Eric BlairSalim S. Abdool KarimGeorge BelovSally RobertsGeorge C RussellSamuel K. CamposGeorge RohrmannSandra Blaise-BoisseauGeorgios VidalakisSandra CiesekGerald HeckelSandra JunglenGérard DemangeatSandra Martínez-TuriñoGerd SutterSandra SkujaGergely TekesSangryeol RyuGerhard StegerSantanu BoseGerhard ThielSao-Jose CarlosGermán MartínezSara MoutaillerGert ZimmerSarah CaddyGhazi KayaliSarah E. JacksonGian Mario CossedduSarah GilbertGian Paolo AccottoSarah LondriganGianvito LanaveSascha TrappGideon MordecaiSatoshi AkagiGil MorSaurabh SrivastavaGilberto SantiagoSaverio PaltrinieriGilda TachedjianSayantan BoseGill ElliottScott D. PeganGilles GadeaScott LovellGiorgio GambinoScott P. KenneyGiorgio PalùScott ReidGiovanna RappoccioloScott W. NelsonGiovanni FranzoSebastian GisderGiovanni IaniroSebastian Maurer-StrohGisselle N. MedinaSebastian VoigtGiulia FranzoniSebastien AlphonseGiulia FreerSébastien PfefferGiulia MatusaliSebastjan RadišekGiulia PezzoniSeema LakdawalaGiuseppe A. SauttoSeiya YamayoshiGiuseppe BertoniSejal ModhaGiuseppe DionisioSek-Man WongGiuseppina SannaSelvarani VimalanathanGlenn F. RallSeokyoung KangGloria ArriagadaSeonghyang SohnGo AtsumiSerena MassariGonzalo MoratorioSerge BénichouGopas JacobSergei BazhanGöran WadellSergei NekhaiGordana Wozniak-KnoppSergey A. AkimovGraham BelshamSergey MorzunovGraham BurgesSergio G. BartualGregory A. NewbySeshi SompuramGregory MelikianSeung-Joo LeeGregory MoseleySeungtaek KimGriffith ParksSeverin O. GudimaGrigorios AmoutziasShan Ali ZaidiGuanghui WuShannon RoncaGuang-Wu ChenShannon WhitmerGuan-zhu HanShaokang ZhangGuido Van MarleSharifah Syed HassanGuido WollmannSharmi ThorGuillaume CastelShauna KaplanGuillermo Hugo Jiménez-AlemánShaw AkulaGunilla B. Karlsson HedestamShawn BabiukGunnel HalldenShawna McCallinGünther SchönrichSheldon MorrisGuoQing LiShelton S. BradrickGus KousoulasShengbo CaoGustavo Del RealSheng-Fan WangGustavo DelhonShenglin MeiGustavo G. GómezShengzhang DongGustavo RomaySherwood CasjensGuy LemayShibo JiangGyőző L. KajánShigeki TakedaH. Joel HutchesonShih-Feng FuH. Michael G. LattorffShih-Yen LoHai LiShin-Hee KimHamed SeifiShinya MaekawaHana M. DobrovolnyShirleen SohHanna M OksanenShitao LiHanna OksanenShivali S. JoshiHans YsselShollie M. FalkenbergHans-Peter HartungShou-jiang GaoHanu PappuShumpei WatanabeHany AnanyShunbin NingHarald BrüssowShunichi UrayamaHector Aguilar-CarrenoShuntai ZhouHee-Chun ChungShuo SuHeejoon MyungShuvojit BanerjeeHeidi DrummerShyan-Song ChiouHeike Schmidt-PosthausSiamak ZohariHeinzpeter SchwermerSiddharth R. KrishnamurthyHelen CrookeSiddhartha DattaHelena J. MaierSidney J. HayesHelena MaierSijun LiuHélène DutartreSilvia FacciniHelene NorderSilvia García-BujalanceHélène SanfaçonSilvia PreziusoHelle Bielefeldt-OhmannSimon CocklinHenk-Jan SchuurmanSimona AnticoliHenry FechnerSimonetta VivianiHenryk CzosnekSimopekka VänskäHerman B. ScholthofSira Abdul RahmanHermann UngerSisko TauriainenHernan Garcia-RuizSiyu ZhuHervé SeligmannSmartt Chelsea T.Heshan Sam ZhouSo NakagawaHetron Mweemba Munang’anduSo Young YooHeung Kyu LeeSonia MediouniHezhao JiSonia ZuñigaHicham El CostaSonu SubudhiHideaki Mabashi-AsazumaSoon Young PaikHideki EbiharaSophie BouvaineHideki KondoSophie ClementHiep VuSophie Zinn-JustinHie-Won HannSotaro ChibaHiroakiSouvik GhoshHiroaki OkamotoSreeKumar BalanHiroaki TokiwaSreekumar OthumpangatHirokazu KimuraSt Patrick ReidHiromitsu MoriyamaSteeve BoulantHisashi AkiyamaStefan PöhlmannHolly J. PophamStefan TaubeHolly R. HughesStefan VilcekHolly SheltonStefan WegerHongmin LiStefania LauziHongping WeiStefania Mirela MangHorst NeveStefania PollastroHuaJi QiuStefanie Christine BeckerHua-Ji QiuStefano AquaroHubert NiestersStefano ButtòHugo OliveiraStefano PetriniHui-Min ChungSteffan T. NawrockiHui-Wen ChangStephan LorenzenHui-Wen ChenStephan PeterHung-Chih YangStephane PronostHung-Yi WuStephane RetyHussin A RothanStephanie DeWitte-OrrHye Won JeongStephanie M LimHyuk-Joon KwonStephanie PfaenderHyunbo ShimStephanie TalkerHyung-Kwan JangStephen C. HardiesIain M. MorganStephen ChallacombeIanStephen J. PriceIan MendenhallStephen LeeIan MolineuxStephen R WelchIgnacio MenaSteve KimbleIgor JurakSteve PetrovskiIgor KoloniukSteven BradfuteIgor LukashevichSteven HagensIgor MorozovSteven M. PascalIgor PaploskiSteven ReidIkuo ShojiSteven RockmanIlhem MessaoudiSteven ShortIlka EngelmannSteven WolfInmaculada FerriolStriker RobInna P. SolyanikovaStuart MacFarlaneIoly Kotta-LoizouSubbroto Kumar SahaIrina Isakova-SivakSue BaigentIrina V. KiselevaSue VandeWoudeIrit DavidsonSue W. LiebmanIsabella EckerleSuh-Chin WuIsabelle Bekeredjian-DingSung Hwan KangIsrael PagánSunil MorIvan KosikSunitha SukumaranIvan KuzminSusan MooreIvo M. FoppaSusan PayneJ. David BeckhamSusana GuixJ. Stephen LodmellSusumu KatsumaJack H.Z. WuSuvi KuivanenJackie MaharSuzanne G M A PasmansJacob S. YountSven PischkeJacob YountSvetlana AntonyukJacques IzopetSylvain De BreyneJacques Le PenduSylvaine BoissinotJae-Ku OemSylvie LecollinatJaime IranzoSylwia BlochJakob ReiserSzecheng LoJakub Dalibor RybkaTadahisa TeramotoJames A. EllisonTadeusz KaczorowskiJames B. WhitneyTae-Jin ChoiJames BinleyTae-Ju ChoJames BullTaisuke HorimotoJames D. BrienTaisun KimJames EvermannTakanobu KatoJames JancovichTakashi AguiJames NgTakashi KimuraJames StrongTakayuki HoshinaJames Van EttenTakele ArgawJames W. GillespieTakeshi KurosuJan ClementTakeshi SaitoJan MünchTamaki Uehara-IchikiJan Philipp Meier-KolthoffTamir TullerJana ProdělalováTanya GolubchikJanet E. MertzTarik GheitJanusz PaweskaTatiana I. SamoylovaJaquelin P. DudleyTatiana TimchenkoJason BlackardTatjana Vilibić-ČavlekJason BodilyTatsufumi UsuiJason BottenTatsuo KandaJason C. HuangTatsuo SuzutaniJason E. ComerTeemu SmuraJason KimataTeiji SawaJason KindrachukTeresa De Los SantosJason MackenzieTero AholaJavier OgemboTero TuomivirtaJavier Orlando CifuenteTerry JonesJavier Sanchez-CespedesTeruo SanoJay R RadkeTetsuo NakayamaJdith WhiteTetsuro IkegamiJean DubuissonTetsuya FuruyaJean François ÉtardTetyana I. VasylyevaJean François MouilletTheresa Li-Yun ChangJean Paul PirnayThérèse CoudercJean RommelaereThibaut CrepinJean-Heinrich DaugroisThomas GallagherJean-Paul PirnayThomas HorvatitsJean-Pierre FrossardThomas KlimkaitJean-Pierre PerreaultThomas MichielsJean-Sebastien CasalegnoThomas P. PeacockJefferson SantosThomas R. FuerstJeffery DeStefanoThomas SmithJeffrey J. HodgsonThomas TuJeffrey RobertsThomas VahlenkampJeffrey WilsonThomas YuillJenner Karlisson Pimenta Dos ReisTian DebinJennifer AltomonteTimo GreinerJennifer CorcoranTimothy GreenJennifer DugganTimothy John MahonyJennifer R. BrumTimothy P. NewsomeJenny WilsonTimothy RoseJens B. BosseTimothy SheahanJens H. KuhnTobias KäserJeremy V. CampTobler KurtJernej JakseTodd G. SmithJérôme GouttenoireTohru SuzukiJesse ArbuckleTohru YanaseJesse BrunnerTomas RumlJessica BelserTomas StrandinJessica Dal ColTomáš VeselýJessica SmithTomasz StenzelJessica SpenglerTomasz ŻokJesus HernandezTommy LamJesús L. RomaldeTomohiro IshikawaJesus RequenaTomoichiro OkaJi Young YooTomomi TakanoJian XuTomoyuki NabeshimaJianfa BaiTony GoldbergJianming QiuTorben Sølbeck RasmussenJiann-Ruey HongTorsten SeuberlichJianqiang ZhangToru TakimotoJianrong LiToufic ElbeainoJian-Wei WangTrevor BraselJiaxin LingTrevor WilliamsJiban Kumar KunduTrina RacineJiewen GuanTristan RenaultJiexiong XieTroy SuttonJill DembowskiTsuge MasatakaJim EvermannTsung-Hsien ChangJim StrongTsutomu KageyamaJimmy DikeakosTsutomu OmatsuJing JinTurhan MarkussenJingen ZhuTzong-Yuan WuJingjing PeiUdo ReichlJingyou YuUlf DittmerJinquan LiUlf PetterssonJin-Wook KimUlrich DesselbergerJiri HejnarUlrich MelcherJishu ShiUlrich SpenglerJitka ForstovaUrs GreberJoachim De MirandaVaibhav TiwariJoakim EsbjörnssonValentina SvicherJoanna ParishValeria LullaJoAnne OliverValérie ChoumetJoão MesquitaVan G. WilsonJocelyne PiretVanessa BarrsJocelyne WalterVanessa Meier-StephensonJody Hobson-PetersVarpu MarjomakiJoe MihaljevicVasanthi AvadhanulaJoe MymrykVeerupaxagouda PatilJoel C. WattsVeit MichaelJoel N. MaslowVelia Ramirez-AmadorJoel RovnakVelmurugan BalaramanJoel S. GriffittsVenugopal NairJohanna B. WithersVera L. TarakanovaJohanna FraserVera V. MorozovaJohanne PoudrierVerónica MartínJohn AlcornVeronica P. CostantiniJohn BinghamVeronica SolovevaJohn DunnVesa HytönenJohn EllisVicky Van SantenJohn FlanneryVictoria K. BaxterJohn HarrisVictoria LawsonJohn HuVida van StadenJohn LednickyVijaykrishna DhanasekaranJohn M. K. RobertsVinay PathakJohn M. LehmanVincent BourretJohn PetterssonVincent MalletJohn T. BatesVineet MenacheryJohn T. PattonVirginie DoceulJohn WhiteViswanath RagupathyJohn-Sebastian EdenVladimir SavicJon SinVladmir ChulanovJonas KlingstromVolker NickeleitJonathan AudetWalid AzabJonathan DennisWalter AtwoodJonathan FogleWanbo TaiJonathan GuitoWataru KamitaniJonathan Paul StoyeWataru MitsuhashiJonathan PerreaultWei LiuJong-Hyun ParkWei ZouJorba JaumeWeifeng GuJörg HofmannWei-Gang HuJorge CarrilloWeiheng SuJorge Gomez-GutierrezWeiran ShenJorma HinkulaWelkin JohnsonJorunn JørgensenWelkin PopeJosé AlmendralWendy MauryJosé D. DebesWendy SpragueJosé G. OlveiraWenxing XuJose L GonzalesWenyu HanJosé L. NievaWerner J. D. OuwendijkJosé Luis AffranchinoWildriss ViranaickenJose María CasasnovasWilliam C. WilsonJose María Rodríguez-CallejaWIlliam DundonJosé Miguel Azevedo-PereiraWilliam FischerJose PenadesWilliam GrantJosep QuerWilliam N. MwangiJosephWilliam S. MasonJoseph Che-Yen WangWilliam SwitzerJoseph DeRisiWim van der PoelJoseph GloriosoWojciech RozekJoseph HughesWolfgang FischerJoseph KendraWon-Il KimJoseph TucciXaolin ChenJoseph ZiegelbauerXaver SewaldJosephine Y. AllerXavier SaelensJoshua MungerXianfang WuJowita Samanta NiczyporykXianglei LiuJoy Y. FengXiangmin LiJoyce JoseXiangmin ZhangJozef NissimovXiao-Fang YuJuan Antonio Diaz PendonXiaohong ShiJuan BarcenaXiaorong TaoJuan C. de la TorreXIAOWEN CHENGJuan ZapataXiao-Wen ChengJuan-Carlos SaizXiaowu PangJudd AikenXiaoying LiuJuditXu TanJudith GottweinXue-Jie YuJuexin WangXueling WuJuhee AhnXufang DengJukka MustonenXun ChenJulia R. ClarkeXuping XieJulià BlancoYael Fridmann-SirkisJulia DinaYa-Fang MeiJulia FrunzkeYa-Juan QianJulia KyddYamada KentaroJulian DruceYan XuJulian LeibowitzYang YangJulian TangYanhua LiJulian W. TangYan-Jang HuangJuliana Freitas-AstúaYanping TianJulie A. HicksYanyan YangJulie LuciforaYasuko MoriJulie PfeifferYasutsugu SuzukiJulie ThomasYechiel ShaiJulio Rodriguez-AndresYen-Hung ChowJun HangYi NiJun InoueYi-Chin FanJun KanekoYijun ZhangJun WangYin XinJunji KawakamiYinfeng ZhangJunji XingYing WangJurg NueschYingyun CaiJürg NüeschYi-Yuan YangJustin A. RobyYize LiJustin G. JulanderYoichi FuruyaJustin Jang Hann ChuYoko MatsuzakiJustin K. HinesYong GaoJustin MeyerYong-Hui ZhengJu-Tao GuoYongli XiaoJyunsuke ShiraiYongming SangKalia S. I. BistolasYoshikazu TanakaKalle SakselaYoshinao KuboKanakala SurapathruduYoshinori NakazawaKang-Seuk ChoiYou-Hee ChoKarel PetrzikYoukyung ChoiKaren BeemonYoung Bong KimKari DebbinkYuchen XiaKarim EssaniYue LiuKarin HolmfeldtYuetsu TanakaKarin KosulinYuji ItoKarin WeberYuki FuruseKariuki NjengaYun Young GoKarl Kai McKinstryYunjeong KimKarla HelbigYu-Shin NaiKarla PassalacquaYusuke MatsumotoKaroly TothYuzuru TaguchiKarthik ChamakuraYves BriersKaryna RosarioYves GaudinKata FarkasZach AdelmanKatalin SalánkiZexu MaKatarzyna Danis-WlodarczykZhan WenbinKatarzyna PodgórskaZhaohua ZhongKatarzyna Ranoszek-SoliwodaZhengli ShiKateri BertranZhenhua ZhengKatherina SewaldZhifang ZhangKatherine BrownZhihong HuKatherine SpindlerZhihui YangKathleen L. HefferonZhilong YangKathleen Laura HefferonZhi-Ming ZhengKathrin SpendierZhongde WangKathryn M. JonesZongdi FengKathryn Marie KauffmanZuanning Yuan

